# Comparative Genomic and Resistance Analysis of ST859-KL19 and ST11 Carbapenem-Resistant *Klebsiella pneumoniae* with Diverse Capsular Serotypes

**DOI:** 10.3390/antibiotics15010040

**Published:** 2026-01-01

**Authors:** Xiao Wang, Jun Feng, Yue Zhang, Ye Qiu, Bowen Yang, Yanru Liang, Yuanping Wang, Bing Zhao, Lili Ren

**Affiliations:** 1 National Institute of Pathogen Biology, Chinese Academy of Medical Sciences & Peking Union Medical College, Beijing 102629, China; wangxiaoyouran@126.com; 2 Shanghai Pudong New Area Center for Disease Control and Prevention (Shanghai Pudong New Area Health Supervision Institute), Shanghai 200136, China; zyuer1997@163.com (Y.Z.); pdcdcxd_qy@163.com (Y.Q.); 18916161522@163.com (B.Y.); lyr1985254806@163.com (Y.L.); lingxi00db@163.com (Y.W.); 3 Shanghai Municipal Center for Disease Control and Prevention, Shanghai 201106, China; fengjun@scdc.sh.cn

**Keywords:** carbapenem-resistant *Klebsiella pneumoniae*, antimicrobial resistance, whole-genome sequencing, ST11, ST859

## Abstract

**Background:** In China, Carbapenem-resistant *Klebsiella pneumoniae* (CRKP) is dominated by sequence type 11 (ST11) harbouring KPC-2, with KL64 displacing KL47 and KL25 emerging. ST859 (ST11 variant) has caused outbreaks, but its epidemiology is unclear. **Materials and Methods:** A total of 99 non-duplicate CRKP isolates were collected from June to December 2024. Antimicrobial susceptibility was determined by broth microdilution. The genomic sequences of the strains were obtained using next-generation sequencing technology. Resistance genes, virulence loci, and plasmid replicons were identified with Kleborate, Abricate, and MOB-suite, respectively. **Results:** ST11 accounted for 63.64% and ST859 for 15.15%. All ST859 were KL19, while ST11 were mainly KL25 (60.32%) and KL64 (26.98%). 76.8% co-harbored carbapenemase and extended-spectrum beta-lactamase (ESBL) genes, with KPC-2 and CTX-M-65 being the predominant types. Susceptibility rates were 100% to tigecycline, and 78.79% to ceftazidime/avibactam. ST859 CRKP isolates exhibited higher phenotypic resistance to tetracycline and colistin than ST11 CRKP isolates (*p* < 0.05), and carrying LAP-2, QnrS1, QnrS10, and *tet*(A) more frequently. ST11-KL25 showed higher resistance to amikacin, gentamicin, and chloramphenicol, with increased prevalence of CTX-M-65, TEM-1, *rmt*B, *cat*A2, and *dfr*A14 compared to ST11-KL64 (*p* < 0.05). IncF was the most prevalent replicon and both ST859 and ST11 CRKP carry conjugative resistance plasmids, and the host range is predominantly Enterobacterales. **Conclusions:** ST859-KL19 ranks second to ST11 with higher resistance to tetracyclines and colistin. ST11-KL25 may have already displaced ST11-KL64 as the predominant capsular type in Shanghai, with distinct resistance profiles between KL variants. Long-term, multicenter surveillance is urgently needed to delineate the evolutionary trajectory and clinical impact of these emerging clones.

## 1. Introduction

Carbapenem-resistant *Klebsiella pneumoniae* (CRKP), the flagship of carbapenem-resistant Enterobacterales (CRE), was placed in the “critical-priority” tier of the 2024 World Health Organization (WHO) Bacterial Priority Pathogens List [1]. Its rapid global dissemination has been accompanied by life-threatening infections with mortality rates that can exceed 50%, underscoring an escalating public health emergency [2]. Colistin and tigecycline are among the last-resort agents for treating CRKP infections. The production of carbapenemases, particularly *Klebsiella pneumoniae* carbapenemase (KPC) and metallo-β-lactamases (MBL), constitutes the primary molecular mechanism of CRKP emergence [3]. In Europe and the United States, the spread of carbapenem resistance is mainly driven by *Klebsiella pneumoniae* sequence type 258 (ST258). In contrast, ST11 has become the main CRKP lineage in China, with KPC-2 being the most common carbapenemase [4,5]. Initially, CRKP was not regarded as hypervirulent, but several fatal outbreaks of severe pneumonia caused by ST11 hypervirulent carbapenem-resistant *Klebsiella pneumoniae* (hvCRKP) have been documented in China [6,7,8], particularly those with capsular types KL64 and KL47, which exhibit extensive antimicrobial resistance and enhanced pathogenicity [5]. These strains typically harbor conjugative IncFII plasmids mediating KPC-2 dissemination [9]. Notably, ST859 is a single-locus variant of ST11, differing only in the housekeeping gene *tonB* while sharing identical alleles at the remaining six loci (*gapA*, *infB*, *mdh*, *pgi*, *phoE*, and *rpoB*). In 2022, an outbreak of ST859-KL19 CRKP was documented at a teaching hospital in China [10]. ST859 CRKP has been reported to develop resistance to ceftazidime/avibactam (CZA) [11]. The ST859 *Klebsiella pneumoniae* strains isolated from peacocks harbor two plasmids carrying multiple resistance genes including *bla*_CTX-M-3_, *aac*(6′)-Ib-cr and *qnr*B91. And these resistance genes are located within a novel mobile composite transposon, Tn7131, which facilitates horizontal gene transfer [12]. Regrettably, compared with the widely studied hypervirulent, multidrug-resistant clones ST11 and ST258, ST859 remains poorly characterized. As of August 2025, a PubMed search combining “ST859” and “*Klebsiella pneumoniae*” yields only three entries, none of which offer systematic phenotypic or genomic characterization. In this study, we conducted a systematic analysis of the antibiotic resistance and virulence of ST859 CRKP isolates, comparing them with the dominant ST11 clone in China to evaluate the potential epidemic risk of ST859.

Adhesive fimbriae, capsular polysaccharide, lipopolysaccharide, and siderophores or iron carriers constitute the principal virulence factors driving *Klebsiella pneumoniae* pathogenicity [3,5]. Compared with strains lacking capsules, strains with capsular polysaccharides exhibit significantly enhanced virulence. Based on capsular antigen diversity, *Klebsiella pneumoniae* has been classified into more than 95 capsular types [13]. The capsular types of ST11 CRKP strains were identified as K2, K9, K16, K19, K31, K39, K47, K57, K62, K64, K80, K102, K103, K105, K125, etc. [14]. Among ST11 CRKP, KL47 and KL64 have successively predominated in China [15]. Surveillance data show that ST11-KL47 was the dominant CRKP lineage prior to 2015, after which ST11-KL64 gradually replaced it in China [16]. This emerging lineage likely arose via genomic recombination from ST11-KL47 strains [17] and exhibits greater virulence than its KL47 counterpart [15]. In recent years, ST11-KL25 strains carrying KPC-2-type carbapenemases and multiple virulence plasmids have been detected in numerous Chinese hospitals, underscoring their potential for widespread dissemination [18,19]. A recent retrospective study in Huai’an City, China, showed that KL64 (56.74%) and KL25 (39.53%) had become the main capsular types of ST11 CRKP isolates [20]. Furthermore, KL25 has become the primarily capsular types prevalent in a tertiary teaching hospital in China [21]. These findings indicate that ST11-CRKP may adapt to environmental selective pressures through capsular switching, yet differences in transmission fitness and resistance profiles among capsular types remain understudied. To address this gap, this study systematically compared ST11 CRKP strains across different capsular types to elucidate their antimicrobial resistance and transmission dynamics, providing molecular evidence for targeted infection control strategies.

## 2. Results

### 2.1. Antibiotic Susceptibility Profiles

**Figure 1:**
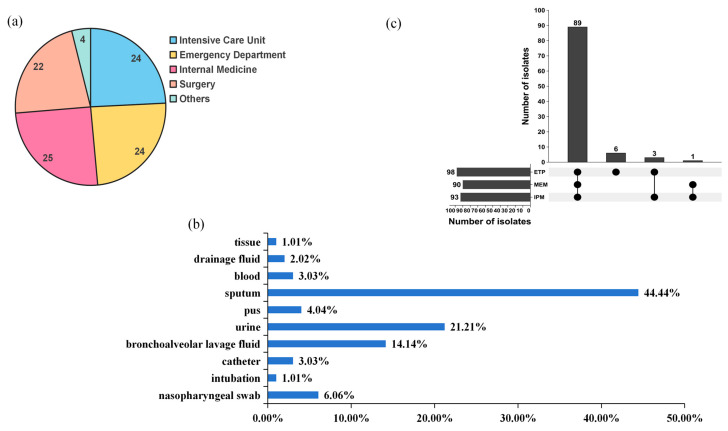
Basic information on 99 CRKP strains. (**a**) Distribution of clinical isolates by department. (**b**) The upset plot shows the resistance of 99 CRKP strains to three different carbapenem antibiotics. Experiments were performed in triplicate with identical results (*n* = 3). (**c**) The bar chart shows the composition ratio of CRKP. MEM, Meropenem; IPM, Imipenem; ETP, Ertapenem.

A total of 99 non-repetitive CRKP isolates were collected from eight hospitals, mainly from internal medicine, surgery, intensive care units (ICU), and emergency departments (Figure 1a, Table S1). These isolates were obtained from patients’ urine, blood, sputum, and other clinical specimens, with sputum accounted for the largest proportion (44.44%, 44/99) (Figure 1b, Table S1). All isolates were resistant to at least one carbapenem antibiotic, including meropenem (MEM), imipenem (IPM), and ertapenem (ETP). Among carbapenem antibiotics, ETP showed the highest resistance rate (98.99%, 98/99), and 89 isolates resistant to IPM, MEM, and ETP simultaneously (Figure 1c). The 99 CRKP isolates showed high susceptibility rates to tigecycline (TIG) (100%, 99/99), streptomycin (STR) (87.88%, 87/99), and ceftazidime/avibactam (CZA) (78.79%, 78/99). Conversely, the resistance rate for ampicillin (AMP) was as high as 100%. The resistance rates for cephalosporins, carbapenems, and quinolones all exceeded 90%. Compared with other antibiotics, the 99 CRKP isolates exhibited high susceptibility to two polymyxin antibiotics, polymyxin B (PB) (7.07%, 7/99) and colistin (CT) (10.10%, 10/99) (Table 1).

### 2.2. Genome Analysis

**Figure 2:**
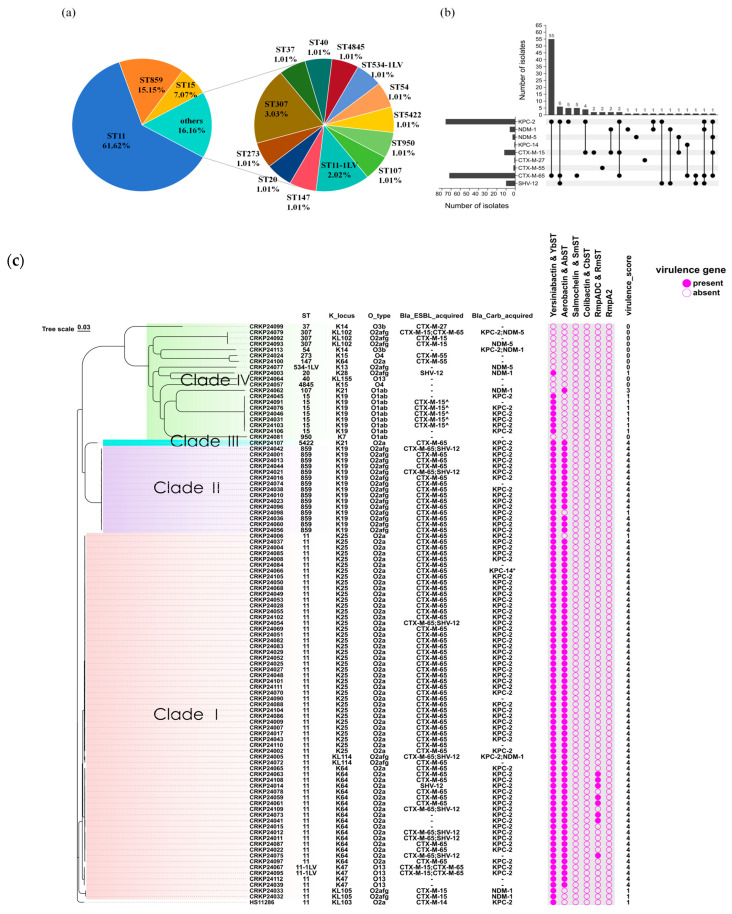
Whole genome analysis of 99 CRKP strains. (**a**) Sequence type distribution of CRKP strains. (**b**) Distribution of carbapenemase and ESBL resistance genes in 99 CRKP strains. (**c**) Genetic features of the 99 CRKP isolates in Pudong, Shanghai. ^ Gene carried on a mobile genetic element (plasmid or transposon), implying potential for horizontal gene transfer. * Functional mutation confirmed. – Not predicted.

Multilocus sequence typing (MLST) resolved 16 STs among the 99 isolates. ST11 predominated (61.62%, 61/99), followed by ST859 (15.15%, 15/99), ST15 (7.07%, 7/99) and various other STs (Figure 2a, Table S2). Two isolates (CRKP24067 and CRKP24095) were designated ST11-1LV by Kleborate. They differ from canonical ST11 only by a partial mismatch in *gapA*, while the other six housekeeping loci are identical. Carbapenemase genes were detected in 83 isolates (83.84%, 83/99), with KPC-2 overwhelmingly predominant (87.95%, 73/83). The CRKP24005 strain detected in urine and the CRKP24113 strain isolated from tissues carried both KPC-2 and NDM-1 enzymes, while the CRKP24079 strain detected in urine carried both KPC-2 and NDM-5 enzymes. ESBL-associated resistance genes were detected in 86 isolates (86.87%, 86/99), predominantly CTX-M-65 (86.75%, 72/83) (Figure 2b).

Phylogenetic analysis separated the strains into four major clades. Clade 1 comprised ST11 and its single-locus variant ST11-1LV, ST11-1LV isolates were merged with ST11 for all downstream analyses. Within Clade 1, ST11 strains were further differentiated into five distinct K types: KL25 (*n* = 38), KL64 (*n* = 17), KL47 (*n* = 2), KL105 (*n* = 2), and KL114 (*n* = 2). ST11-1LV strains were associated with the KL47 and O13. Clade 2 consisted exclusively of ST859-KL19. Clade 3 contained ST5422. ST15 and nine additional STs were grouped into Clade 4. For the O-locus, six variants were detected, with O2a predominating (57.58%, 57/99). All ST859-KL19 strains in clade 2 were typed as O2afg, whereas ST11-KL25 and ST11-KL64 strains were both typed as O2a. Notably, ST859 is a single-locus variant of ST11, indicating a close phylogenetic relationship. Two ST11-KL105 isolates (CRKP24032 and CRKP24033) carried NDM-1 carbapenemase but exhibited lower virulence (virulence score = 1) (Figure 2c).

### 2.3. Comparison of Antimicrobial Resistance Between ST859 and ST11 CRKP

**Figure 3:**
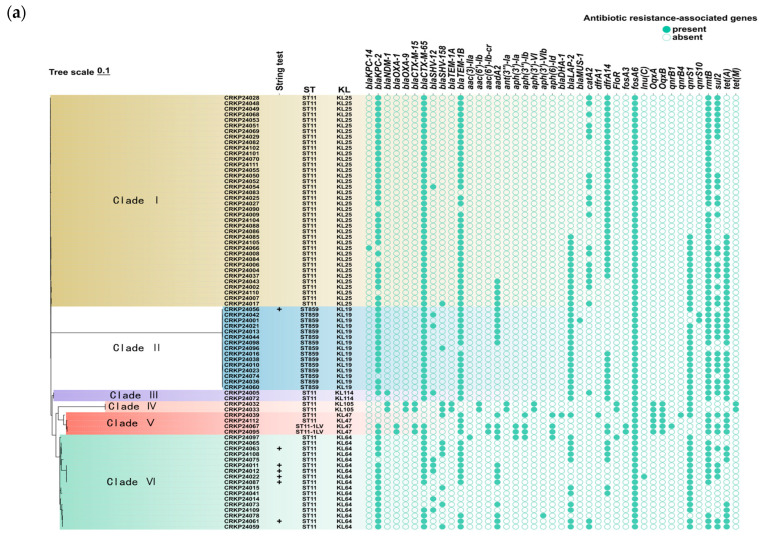

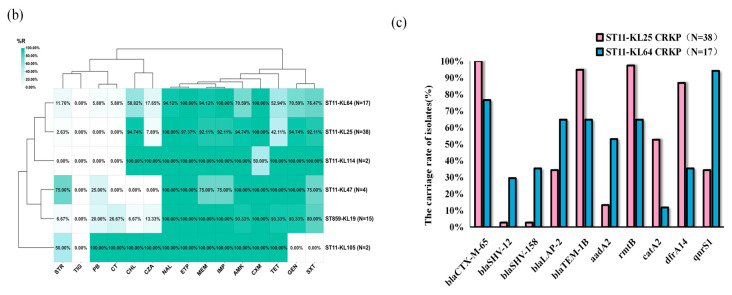
Comparison of pathogenicity and antimicrobial resistance between ST859 and ST11 CRKP. (**a**) Analysis of antibiotic resistance gene in ST859 and ST11 CRKP isolates. “+” indicates a mucus filament > 5 mm, denoting a highly mucoid phenotype. (**b**) Antimicrobial resistance rates between ST859 and ST11 CRKP isolates to different antimicrobial agents. (**c**) Statistical analysis of the presence of resistance genes in ST11-KL25 and ST11-KL64 CRKP isolates.

Among the ST857 and ST11 CRKP isolates, one ST859 CRKP (CRKP24098) and three ST11 CRKP strains (CRKP24006, CRKP24032, and CRKP24033) exhibited low virulence (virulence score = 1), while the remaining strains demonstrated high virulence (virulence score = 4) (Figure 2c). Fisher’s exact test was used to compare antibiotic-resistance rates between the two strain groups. As shown in Table S3, all ST859 and ST11 CRKP isolates were 100% resistant to AMP, ampicillin/sulbactam (SAM), cefoxitin (CFX), cefazolin (CFZ), cefotaxime (CTX), ceftazidime (CAZ), cefepime (CPM) and ciprofloxacin (CIP), but universally susceptible to TIG. Compared with ST11 CRKP isolates, ST859 CRKP isolates displayed significantly higher resistance to tetracycline (TET) and CT, but lower resistance to chloramphenicol (CHL) (*p* < 0.05) (Table 2). Further comparison of antibiotic resistance profiles between ST859 CRKP isolates and ST11 CRKP isolates with different capsular types revealed that ST11-KL105 CRKP isolates exhibited the highest resistance. However, continued surveillance is required to confirm this finding as the sample size was extremely small (*n* = 2). The resistance profiles were similar between ST859-KL19 and ST11-KL47 isolates, as well as between ST11-KL25 and ST11-KL64 isolates. Notably, ST11-KL25 exhibited significantly higher resistance to amikacin (AMK), gentamicin (GEN), and CHL than ST11-KL64 CRKP isolates (*p* < 0.05) (Figure 3b, Table 3).

A total of 15 distinct resistance genes were identified among the 15 ST859 CRKP isolates, whereas 39 different resistance genes were detected in the 63 ST11 CRKP isolates. All isolates harbored the *fosA6* gene. Compared with ST11 CRKP isolates, ST859 CRKP isolates exhibited higher carriage rates of *bla*_LAP-2_, *qnr*S1, *qnr*S10, and *tet*(A), but a significantly lower prevalence of *cat*A2 (*p* < 0.05) (Table S4). The genes *bla*_OXA-1_, *bla*_OXA-9_, *bla*_TEM-1A_, *aac*(6′)-Ib, *aac*(6′)-Ib-cr, *ant*(3″)-Ia, *aph*(3′)-VI, and *tet*(M) were exclusively detected in ST11-KL105 isolates. *Bla*_NDM-1_ was found only in ST11-KL114 and ST11-KL105 CRKP isolates (Figure 3a). Further analysis revealed that ST11-KL25 isolates had significantly higher carriage rates of *bla*_CTX-M-65_, *bla*_TEM-1B_, *rmt*B, *cat*A2, and *dfr*A14 than ST11-KL64 isolates, while the prevalence of *bla*_SHV-12_, *bla*_SHV-158_, *bla*_LAP-2_, *aad*A2, and *qnr*S1 was lower in ST11-KL25 (*p* < 0.05) (Figure 3c, Table S5).

### 2.4. Plasmid Profiling of ST859 and ST11 CRKP

**Figure 4:**
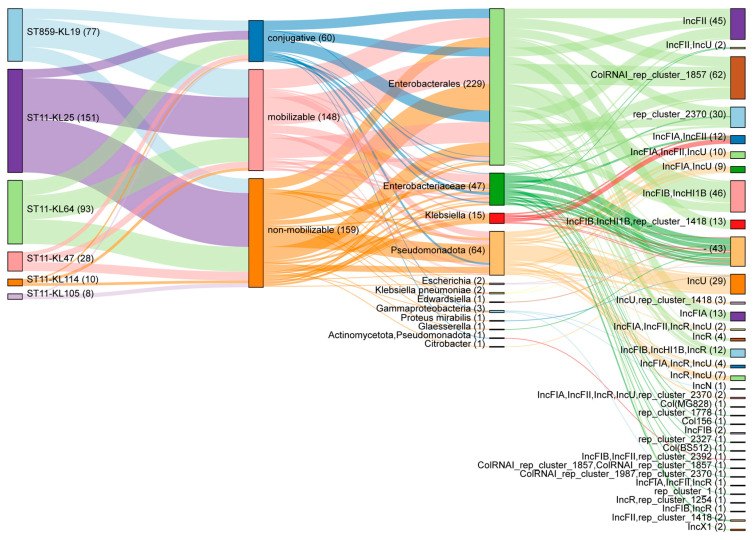
Genomic analysis of plasmids in ST859 and different capsular serotype ST11 CRKP isolates. Sankey diagram illustrating the association between plasmid type, predicted host range, and plasmid replicon type in ST859-KL19, ST11-KL25, ST11-KL64, ST11-KL47, ST11-KL105 and ST11-KL114 CRKP isolates. The thickness of each line is proportional to the number of plasmids.

To assess the co-localization risk of virulence and resistance genes on plasmids, we systematically characterized the plasmid repertoire of 78 ST859 and ST11 CRKP isolates using Mobsuite v3.19 (MOB-recon module). A total of 367 plasmids (0–10 per isolate) were predicted across the 78 isolates, spanning six known replicon families (Col, IncN, IncU, IncF, IncH, and IncR), with IncF being most prevalent (Figure 4). The MOB-typer module further predicted the conjugative potential and host range of the plasmids, identifying 60 conjugative, 148 mobilizable, and 159 non-mobilizable plasmids. Among conjugative plasmids, IncFII predominated (52/60, 86.87%), and the predicted host range was primarily restricted to Enterobacterales (229/367, 62.40%) (Figure 4). Of the 60 conjugative plasmids, 56 (93.33%) carried antibiotic resistance genes, such as TEM-1, KPC-2, and *dfr*A14. Specifically, four ST11-KL64 and one ST859-KL19 CRKP isolates harbored KPC-2 carbapenemase-resistant conjugative plasmid, while two ST11-KL105 isolates carried NDM-1-encoding conjugative plasmids (Table S6). No conjugative plasmid was found to carry both antibiotic-resistance and virulence genes simultaneously. Notably, the virulence genes *iutA*, *iucC*, *iucB*, and *iucA* were found on a conjugative plasmid only in strain CRKP24097 (ST11-KL64) (Table S7). In the other 77 strains, these four virulence genes were exclusively located on non-mobilizable plasmids.

## 3. Discussion

*Klebsiella pneumoniae* has emerged as a major threat due to the convergence of multidrug resistance and hypervirulence [22]. Carbapenem resistance now severely limits treatment options for CRKP infections, posing an urgent clinical challenge [22]. Based on seven housekeeping genes (*gapA*, *infB*, *mdh*, *pgi*, *phoE*, *rpoB*, *tonB*), MLST classifies *Klebsiella pneumoniae* into thousands of sequence types. Globally, ST258 dominates in Europe and North America, whereas ST11, the single-locus variant of ST258 at *tonB*, predominates in China [23,24], consistent with our findings. Notably, we identified ST859, a single-site variant of ST11 differing only at the *tonB* locus, has become the second most common CRKP lineage in our region. All 15 ST859 isolates belonged to capsular type KL19, a prevalence far higher than the sporadic reports in earlier literature [25]. Although surveillance suggests a potential shift from ST11 to ST15 in China [26], ST15 accounted for only 7.07% (7/99) of our collection, whereas ST859 comprised 15.15% (15/99). These findings suggest that CRKP may adapt to selective pressure through clonal succession and capsular switching, and that its molecular epidemiological evolution exhibits geographical heterogeneity. Unique clonal selective pressures may arise from regional differences in antimicrobial usage patterns, patient demographics, and transmission networks.

The most important mechanism underlying carbapenem resistance in CRKP is the production of carbapenemases, including class A carbapenemases (mainly *Klebsiella pneumoniae* carbapenemase, KPC), class B metallo-β-lactamases (mainly New Delhi metallo-β-lactamase, NDM), and some class D OXA-48-like carbapenemases (mainly OXA-181, OXA-232, and OXA-163) [27]. In this study, 83.84% (83/99) of the CRKP isolates carried a carbapenemase gene, with KPC-2 predominating (87.95%, 73/83). Moreover, 75.76% (75/99) of the strains simultaneously harboured the KPC-2 carbapenemase gene and ESBL determinants such as CTX-M-65, constituting “super-resistant” isolates that further restrict therapeutic options beyond CZA. The remaining 16.16% (16/99) of CRKP isolates lacked any detectable carbapenemase genes (NC-CRKP). Despite lacking carbapenemase genes, NC-CRKP mortality was comparable to that of carbapenemase-producing CRKP (C-CRKP), and the significant disease burden it imposes should not be overlooked [28]. Additionally, three strains simultaneously harbored KPC-2 and NDM-1/5, exhibiting resistance to MEM, ETP, IMP, and the novel β-lactamase inhibitor combination CZA, posing a threat to clinical antimicrobial therapy. Fortunately, CRKP strains still exhibit high susceptibility to PB (7.07%, 7/99) and CT (10.10%, 10/99), preserving these as crucial therapeutic options.

The convergence of hypervirulence and carbapenem resistance in *Klebsiella pneumoniae* poses a critical global health threat [22], yet lacks a consensus definition for hvKP identification [29]. While *iucA* is a reliable genetic marker for hvKP [30], all CRKP isolates in this study with a virulence score of 4 and harboring *iucA* lacked additional siderophore genes (e.g., *iroB*). Archetypal hvKP clones (ST23, ST86, ST65) and ST231 typically carry the aerobactin operon (*iuc*) [31] with virulence scores ≥ 3 [32] and our virulence-enhanced isolates were similarly restricted to phylogenetic clades 1–3 (mainly ST859 and ST11 CRKP). However, only seven isolates exhibited a hypermucoviscous phenotype (>5 mm mucus filament), including six ST11-KL64 and one ST859-KL19 CRKP isolates. As literature cautions, the presence of hvKP-associated virulence genes or a hypermucoviscous phenotype does not guarantee a high-virulence phenotype [33]. For CRKP strains, the term “hypervirulence” requires corroboration from mouse or other mammalian infection models [34]. Nevertheless, the high prevalence of *iucA*-positive CRKP remains concerning. Phylogenetic analysis revealed that ST859 CRKP strains exhibited significantly higher resistance rates to TET and polymyxin compared to ST11 CRKP strains, and the carriage rates of LAP-2, QnrS1, QnrS10 and *tet*(A) were likewise markedly elevated. Notably, no plasmid-mediated colistin resistance genes (e.g., *mcr*-1) were detected. However, one ST859 CRKP isolate carried an *mgr*B mutation. This chromosomal mutation can confer colistin resistance through PhoPQ system dysregulation, indicating that ST859 CRKP may acquire resistance via chromosomal mechanisms rather than plasmid acquisition [35]. Polymyxin monotherapy should be used with caution in areas with high prevalence of ST859 CRKP strains, and alternative agents such as TIG or CZA should be considered. Furthermore, the presence of resistance genes such as LAP-2, QnrS1 and *tet*(A) on the same conjugative plasmid not only facilitated transfer between different *Klebsiella pneumoniae* strains but also suggested potential transfer to other *Enterobacteriaceae*. Combined with the broader resistance profile of ST859 CRKP, this suggests that this clone may possess enhanced adaptive advantages. Additionally, all ST859 and ST11 CRKP isolates carried truncated OmpK35 together with OmpK36 harbouring a GD insertion in the L3 loop. This insertion narrows its pore and restricts the diffusion of antibiotics such as carbapenems, thus enhancing resistance.

Capsular polysaccharide is a critical antigen of *Klebsiella pneumoniae*, Surveillance data indicate that ST11-KL47 was predominant in China prior to 2015, after which ST11-KL64 gradually replaced its dominant position [16]. Our data now indicate that ST11-KL25 has become the most prevalent capsular type. Combined with a recent report from China [21], suggest that ST11 CRKP is undergoing continuous micro-evolution and dissemination, with KL25 may be emerging as a new dominant clone. However, whether this shift is region-specific demands long-term, multicentre surveillance. Phylogenetic analysis of ST859 CRKP and ST11 serotype-different strains shows that ST859-KL19 clusters most closely with ST11-KL47/64 CRKP, suggesting that the capsular synthesis gene cluster (cps locus) may spread among different ST-type strains via horizontal transfer or recombination events. Phenotypically, ST859-KL19 CRKP clustered with ST11-KL47, with PB resistance rate exceeding 25%. Colistin and CZA are the last-line agents for CRKP infections in China [36], which implies that the efficacy of polymyxin antibiotics, regarded as the “last line of defense”, may significantly diminish against ST859-KL19 CRKP strains. Regrettably, systematic global research on this clone remains limited, with prior studies offering only fragmented data on virulence and resistance profiles. Comprehensive genomic–clinical–prognostic correlations are still lacking. Finally, we compared the antimicrobial resistance profiles and evolutionary relationships among ST11 CRKP isolates with different capsular types. The results show that the ST11-KL25 isolate exhibited significantly higher resistance to AMK, GEN, and CHL antibiotics compared to ST11-KL64, and the prevalence of resistance genes CTX-M-65, TEM-1, *rmt*B, *cat*A2, and *dfr*A14 were also significantly higher than in ST11-KL64 CRKP isolates, suggesting that ST11-KL25 CRKP may possess a broader resistance spectrum.

This study has several limitations. First, the virulence phenotypes of the included strains and the conjugative transferability of the plasmids were not experimentally validated. We relied solely on functional predictions from bioinformatics tools such as Kleborate and mobsuite, rather than verification through mammalian infection models and conjugation experiments. Second, corresponding clinical outcome data were unavailable for the enrolled patients, including detailed information on treatment regimens, disease progression, and mortality rates. This deficiency precluded a comprehensive correlation analysis between strain-specific characteristics (e.g., capsular serotype, resistance genotype) and clinical prognostic indicators. Addressing these critical gaps through integrated genomic-clinical investigations will be a priority for our future research.

## 4. Materials and Methods

### 4.1. Isolation and Identification of Carbapenem-Resistant Klebsiella pneumoniae

We conducted a retrospective analysis of 99 non-duplicate CRKP strains isolated from various clinical specimens (blood, sputum, urine, etc.) of patients admitted to multiple hospitals in Shanghai, China, between June and December 2024. All strains were collected from inpatient cases by each participating hospital using a sampling strategy of randomly selecting 5–10 strains per month, and all isolates exhibited a carbapenem-resistant phenotype defined by a minimum inhibitory concentration (MIC) ≥ 4 μg/mL for imipenem or meropenem, or ≥2 μg/mL for ertapenem according to the Clinical and Laboratory Standards Institute (CLSI) 34th Edition breakpoints [37]. Detailed epidemiological data, including specimen sources and patient demographics, are provided in Supplementary Table S1.

### 4.2. Antimicrobial Susceptibility Testing

Antimicrobial susceptibility testing of the CRKP isolates was performed by broth microdilution using the AST Panel for Aerobic Gram-Negative Bacilli (Shanghai Fosun Biotechnology Co., Ltd., Shanghai, China). The following 23 agents were tested at the indicated concentration ranges: ampicillin (AMP, 1–64 µg/mL), colistin (CT, 0.125–8 µg/mL), cefazolin (CFZ, 0.5–32 µg/mL), cefotaxime (CTX, 0.25–16 µg/mL), cefoxitin (CFX, 1–64 µg/mL), cefepime (CPM, 1–64 µg/mL), cefuroxime (CXM, 0.5–32 µg/mL), ceftazidime/avibactam (CZA, 0.25/4–8/4 µg/mL), ceftazidime (CAZ, 0.5–32 µg/mL), imipenem (IMP, 0.25–8 µg/mL), ertapenem (ETP, 0.25–8 µg/mL), trimethoprim/sulfamethoxazole (SXT, 0.25–8 µg/mL), ampicillin/sulbactam (SAM, 1/0.5–64/32 µg/mL), nalidixic acid (NAL, 2–64 µg/mL), chloramphenicol (CHL, 2–64 µg/mL), tetracycline (TET, 1–32 µg/mL), tigecycline (TIG, 0.25–8 µg/mL), gentamicin (GEN, 1–32 µg/mL), ciprofloxacin (CIP, 0.015–32 µg/mL), amikacin (AMK, 2–64 µg/mL), polymyxin B (PB, 0.125–4 µg/mL), meropenem (MEM, 0.125–4 µg/mL), streptomycin (STR, 4–32 µg/mL). Results were interpreted as susceptible (S), intermediate (I), or resistant (R) according to the Clinical and Laboratory Standards Institute (CLSI) 34th Edition breakpoints [37]. The *E. coli* ATCC^®^ 25922 and *Klebsiella pneumoniae* ATCC^®^ 700603 strains served as the quality-control strains. Extended-spectrum β-lactamase-producing *K. pneumoniae* (ESBL-KP) was defined as an isolate exhibiting a ceftazidime or cefotaxime MIC ≥ 2 μg/mL that decreases by ≥3 two-fold dilutions in the presence of clavulanate [22]. CRKP was defined as any strain resistant to at least one of IMP, MEM, or ETP. Multidrug-resistant (MDR) strains were those non-susceptible to at least three different antimicrobial classes and pandrug-resistant (PDR) strains was defined as non-susceptibility to all agents in all antimicrobial categories [38].

### 4.3. String Test

A single colony is inoculated onto a blood agar plate and incubated at 37 °C overnight. The bacterial colony was gently pulled up with the inoculation loop and repeated for 3 times. If viscous strings were formed three times and their lengths were longer than 5 mm, the string test was positive and classified as hypermucoid. This hypermucoviscous phenotype is one of the hallmarks of pathogenicity and is used for the preliminary assessment of the pathogenic potential of the strain [39].

### 4.4. Whole Genome Sequencing, Assembly, and Annotation

CRKP isolates stored at −80 °C were recovered and purified on the blood agar plate, and 1~3 pure colonies were scraped with a sterile inoculation ring and transferred into the eppendorf (EP) tubes containing 500 μL of saline to create a bacterial suspension. Genomic DNA was extracted using a magnetic bead adsorption method according to the nucleic acid extraction kit (Tianlong Technology Co., Xi’an, China). For DNA samples with a concentration ≥ 10 ng/μL quantified using a Qubit fluorometer (ThermoFisher Scientific, Waltham, MA, USA), library construction was carried out according to the manufacturer’s instructions (Shanghai BioGerm Medical Biotechnology Co., Ltd., Shanghai, China). High-throughput sequencing was conducted on the MGI DNBSEQ-T7 platform (MGI, Shenzhen, China) with a paired-end 150 bp (PE150) configuration at an average depth of 200×. Only runs yielding ≥ 1 Gb of data with a Q30 ratio exceeding 85% were retained for downstream bioinformatic analysis, ensuring both the reliability and depth of the resulting genomic data. FastQC (v0.11.9) and Fastp (v0.23.2) tools were used for data quality control and removal of low-quality reads and splice sequences. De novo assembly of the filtered data was performed with SPAdes (v3.15.5). The assembled genomes were approximately 5.28~5.97 Mb in size and were annotated with Kraken2 (v2.1.2) to ensure that all subsequent analyses were restricted to the target microorganism.

### 4.5. Bioinformatic and Phylogenetic Analyses

Sequence type (ST), capsule (K-locus), lipopolysaccharide (O-locus), virulence genes, and antimicrobial-resistance genes were performed with Kleborate (v2.1.14) [32] and subsequently verified by Blastn searches against the NCBI nucleotide database (https://blast.ncbi.nlm.nih.gov/Blast.cgi, accessed on 15 October 2025). Kleborate is a dedicated bioinformatic tool for assessing the resistance and virulence of *Klebsiella pneumoniae*. It assigns a resistance score of 0~4 based on the presence/absence of resistance genes such as carbapenemase, and a virulence score of 0~5 according to the detected virulence loci [32] (Tables S8 and S9). For phylogenetic analysis, all genomes were aligned to the reference strain HS22286 (GenBank GCA_000240185.2) using Parsnp [40] (v2.1.4). A maximum-likelihood tree was inferred from the core-genome SNPs and rendered with the ChiPlot web platform [41]. All tools were executed with their default settings. Sequences were deposited to NCBI website under the Bioproject PRJNA1301532.

### 4.6. Determining Plasmid Mobility

Plasmid sequences were de novo reconstructed from assembled genomes with MOB-Suite [42] (v3.19). The MOB-typer module was used to assign relaxase (MOB) and replicon types, generate MOB-cluster codes, and infer putative host range. Mobility was predicted by inspecting three key loci: relaxase (MOB), mating-pair-formation (MPF) genes, and the origin of transfer (oriT). Following the MOB-Suite convention, plasmids were classified as (i) conjugative when both MOB and MPF were present, (ii) mobilizable when relaxase or oriT is present but MPF is absent, and (iii) non-mobilizable when neither MOB nor oriT was found. Antimicrobial-resistance and virulence genes carried by each reconstructed plasmid were subsequently screened with ABRicate (v1.0.1). All analyses were run with default parameters.

### 4.7. Statistical Analysis

SPSS statistical software (v27.0) and GraphPad Prism (v10.3.1) was used for data analyses. Comparisons between groups were conducted using the chi-square test or Fisher’s exact test. Two-sided and *p* < 0.05 was considered to be statistically significant.

## 5. Conclusions

This study provides a comprehensive analysis of the molecular epidemiology, resistance mechanisms, and virulence characteristics of CRKP in Shanghai, China. Results show that CRKP is undergoing continuous clonal evolution. Although ST11 remains dominant, micro-evolutionary subtypes such as ST11-1LV are emerging, and the single-locus variant ST859 has become the second most prevalent lineage in the region. ST859-KL19 CRKP exhibits significantly higher resistance to tetracycline and colistin than ST11 CRKP isolates and frequently harbors conjugative resistance plasmids. Phylogenetically, ST859-KL19 is closely related to the predominant clone ST11-KL64/47 CRKP isolates. Given the lack of systematic global research on ST859, to our knowledge, this represents the first systematic comparison of ST11 and ST859 in the context of CRKP. Furthermore, analysis of resistance patterns across ST11 capsular types indicated that ST11-KL25 may have already displaced ST11-KL64 as the predominant capsular type in Shanghai. Long-term, multicenter surveillance is urgently needed to delineate the evolutionary trajectory and clinical impact of these emerging clones.

## Figures and Tables

**Table 1:** Antibiotic susceptibility profiles of 99 clinical CRKP strains.

Categories of Antibiotics		Antibiotics	No.	%R	No.	%I	No.	%S
β-lactams	Penicillins	Ampicillin	99	100.00%	0	0.00%	0	0.00%
		Ampicillin/sulbactam	97	97.98%	1	1.01%	1	1.01%
	Cephalosporins	Ceftazidime/avibactam	21	21.21%	0	0.00%	78	78.79%
		Cefoxitin	98	98.99%	0	0.00%	1	1.01%
		Cefuroxime	96	96.97%	0	0.00%	3	3.03%
		Ceftiofur	96	96.97%	1	1.01%	2	2.02%
		Cefazolin	98	98.99%	1	1.01%	0	0.00%
		Ceftazidime	97	97.98%	0	0.00%	2	2.02%
		Cefepime	97	97.98%	1	1.01%	1	1.01%
	Carbapenems	Imipenem	93	93.94%	2	2.02%	4	4.04%
		Ertapenem	98	98.99%	0	0.00%	1	1.01%
		Meropenem	90	90.91%	3	3.03%	6	6.06%
Aminoglycosides		Amikacin	78	78.79%	3	3.03%	18	18.18%
		Streptomycin	12	12.12%	0	0.00%	87	87.88%
		Gentamicin	82	82.83%	1	1.01%	16	16.16%
Quinolons		Ciprofloxacin	97	97.98%	1	1.01%	1	1.01%
		Nalidixic acid	95	95.96%	0	0.00%	4	4.04%
Sulfonamides		Trimethoprim/sulfamethoxazole	80	80.81%	0	0.00%	19	19.19%
Tetracyclines		Tigecycline	0	0.00%	0	0.00%	99	100.00%
		Tetracycline	58	58.59%	10	10.10%	31	31.31%
Polypeptide		Polymyxin B	7	7.07%	92	92.93%	0	0.00%
		Colistin	10	10.10%	89	89.90%	0	0.00%
		Chloramphenicol	62	62.63%	18	18.18%	19	19.19%

**Table 2:** Comparison of antimicrobial resistance rates between ST11 CRKP and its single-locus variant ST857 CRKP clinical isolates.

Antimicrobial Agent	ST11 CRKP (*n* = 63)		ST859 CRKP (*n* = 15)		*p* Value
	No.	%R	No.	%R	
Ampicillin	63	100.00%	15	100.00%	/
Ampicillin/sulbactam	63	100.00%	15	100.00%	/
Ceftazidime/avibactam	10	15.87%	2	13.33%	1.000
Cefoxitin	63	100.00%	15	100.00%	/
Cefuroxime	62	98.41%	15	100.00%	1.000
Ceftiofur	63	100.00%	15	100.00%	/
Cefazolin	63	100.00%	15	100.00%	/
Ceftazidime	63	100.00%	15	100.00%	/
Cefepime	63	100.00%	15	100.00%	/
Imipenem	59	93.65%	15	100.00%	0.726
Ertapenem	62	98.41%	15	100.00%	1.000
Meropenem	58	92.06%	15	100.00%	0.577
Amikacin	56	88.89%	14	93.33%	0.971
Streptomycin	7	11.11%	1	6.67%	0.971
Gentamicin	54	85.71%	14	93.33%	0.716
Ciprofloxacin	63	100.00%	15	100.00%	/
Nalidixic acid	62	98.41%	15	100.00%	1.000
Trimethoprim/sulfamethoxazole	53	84.13%	12	80.00%	1.000
Tigecycline	0	0.00%	0	0.00%	/
Tetracycline	33	52.38%	14	93.33%	**0.004** *
Polymyxin B	4	6.35%	3	20.00%	0.246
Colistin	3	4.76%	4	26.67%	**0.030** *
Chloramphenicol	50	79.37%	1	6.67%	**<0.001** *

* Bold value, *p* < 0.05, /, Statistical analysis was not performed as the resistance rates were identical.

**Table 3:** Comparison of antimicrobial resistance rates between ST11-KL64 and ST11-KL25 CRKP clinical isolates.

Categories of Antibiotics		Antibiotics	ST11-KL64 CRKP (*n* = 17)		ST11-KL25 CRKP (*n* = 38)		*p* Value
			No.	%R	No.	%R	
β-lactams	Penicillins	Ampicillin	17	100.00%	38	100.00%	/
		Ampicillin/sulbactam	17	100.00%	38	100.00%	/
	Cephalosporins	Ceftazidime/avibactam	3	17.65%	3	7.89%	0.546
		Cefoxitin	17	100.00%	38	100.00%	/
		Cefuroxime	17	100.00%	38	100.00%	/
		Ceftiofur	17	100.00%	38	100.00%	/
		Cefazolin	17	100.00%	38	100.00%	/
		Ceftazidime	17	100.00%	38	100.00%	/
		Cefepime	17	100.00%	38	100.00%	/
	Carbapenems	Imipenem	17	100.00%	35	92.11%	0.583
		Ertapenem	17	100.00%	37	97.37%	1.000
		Meropenem	16	94.12%	35	92.11%	1.000
Aminoglycosides		Amikacin	12	70.59%	36	94.74%	**0.041** *
		Streptomycin	2	11.76%	1	2.63%	0.462
		Gentamicin	12	70.59%	36	94.74%	**0.041** *
Quinolons		Ciprofloxacin	17	100.00%	38	100.00%	/
		Nalidixic acid	16	94.12%	38	100.00%	0.677
Sulfonamides		Trimethoprim/sulfamethoxazole	13	76.47%	35	92.11%	0.242
Tetracyclines		Tigecycline	0	0.00%	0	0.00%	/
		Tetracycline	9	52.94%	16	42.11%	0.456
Polypeptide		Polymyxin B	1	5.88%	0	0.00%	0.677
		Colistin	1	5.88%	0	0.00%	0.677
Chloramphenicols		Chloramphenicol	10	58.82%	36	94.74%	**0.003** *

* Bold value, *p* < 0.05, /, Statistical analysis was not performed as the resistance rates were identical.

## Data Availability

Sequences were deposited to NCBI website under the Bioproject PRJNA1301532. Please contact author for other data requests.

## Supplementary Materials

The following supporting information can be downloaded at: https://www.mdpi.com/article/10.3390/antibiotics15010040/s1, Table S1: Detailed clinical information of 99 CRKP isolates; Table S2: Detailed genomic characteristics of the 99 CRKP isolates in this study; Table S3: Minimal inhibitory concentration (MIC) for antimicrobials of 99 CRKP isolates; Table S4: Comparison of the presence of resistance genes in ST11 and ST859 CRKP clinical isolates; Table S5: Comparison of the presence of resistance genes in ST11-KL25 and ST11-KL64 CRKP clinical isolates; Table S6: Antimicrobial resistance and virulence genes among the conjugative plasmids; Table S7: Distribution of virulence genes on plasmids carried by clinical isolates of ST859 and ST11 CRKP; Table S8: Definition and explanation of virulence scores in the Kleborate tool; Table S9: Definition and explanation of resistance score in the Kleborate tool.
